# A Superior Squeeze: Superior Vena Cava Syndrome Secondary to Small Cell Lung Cancer

**DOI:** 10.7759/cureus.61717

**Published:** 2024-06-05

**Authors:** Anvit D Reddy, Oshin Rai, Natalie Shaykh, Falguni Patel, Niyati Patel, Ghania Masri

**Affiliations:** 1 Internal Medicine, University of Florida College of Medicine – Jacksonville, Jacksonville, USA

**Keywords:** facial edema, tobacco adverse effects, interventional radiology stent placement, lung cancer surveillance, lung cancer, venography, venoplasty, pulmonary embolism (pe), small-cell lung carcinoma, superior vena cava (svc) syndrome

## Abstract

Superior vena cava (SVC) syndrome is an uncommon yet potentially fatal syndrome occurring after intrinsic or extrinsic compression to the SVC. While there are multiple emerging etiologies for this phenomenon, malignancy remains the most common. It is characterized by several symptoms including facial swelling, extremity swelling, shortness of breath, and headaches. We present the case of a 59-year-old female with a past medical history of cocaine abuse who was admitted for upper extremity swelling and facial edema. Imaging revealed a right suprahilar mass compressing a branch of the right pulmonary artery and SVC, in addition to bilateral segmental and subsegmental pulmonary emboli. She underwent an emergent biopsy and SVC stenting, with immunostaining revealing small cell lung cancer (SCLC). This case highlights a severe presentation of SVC syndrome caused by previously undetected SCLC.

## Introduction

Superior vena cava (SVC) syndrome is an uncommon condition with about 15,000 cases in the United States per year [1]. It is the result of partial or complete obstruction of blood flow returning to the SVC and can be life-threatening with significant morbidity and mortality. The SVC is a thin-walled, low-pressure system that can be compressed intrinsically and extrinsically in the middle or anterior mediastinum. Although the most common cause used to be infectious, such as syphilitic aortic aneurysm and tuberculosis, malignancy is now responsible for more than 90% of cases [2]. The most common malignancy is non-small cell lung cancer (NSCLC) (50%), followed by small cell lung cancer (SCLC) (25%) and lymphomas (10%). Benign causes are rising with intrinsic etiologies of SVC syndrome including thrombi, stenosis, and fibrosis from medical devices such as indwelling catheters and pacemaker leads, hypercoagulable states, or vasculitis [3,4]. 

## Case presentation

A 59-year-old female with a past medical history of diabetes mellitus, coronary artery bypass graft, and cocaine abuse presented to the emergency department (ED) with worsening headaches, shortness of breath, and upper and lower extremity swelling over the past two weeks. 

**Figure 1:**
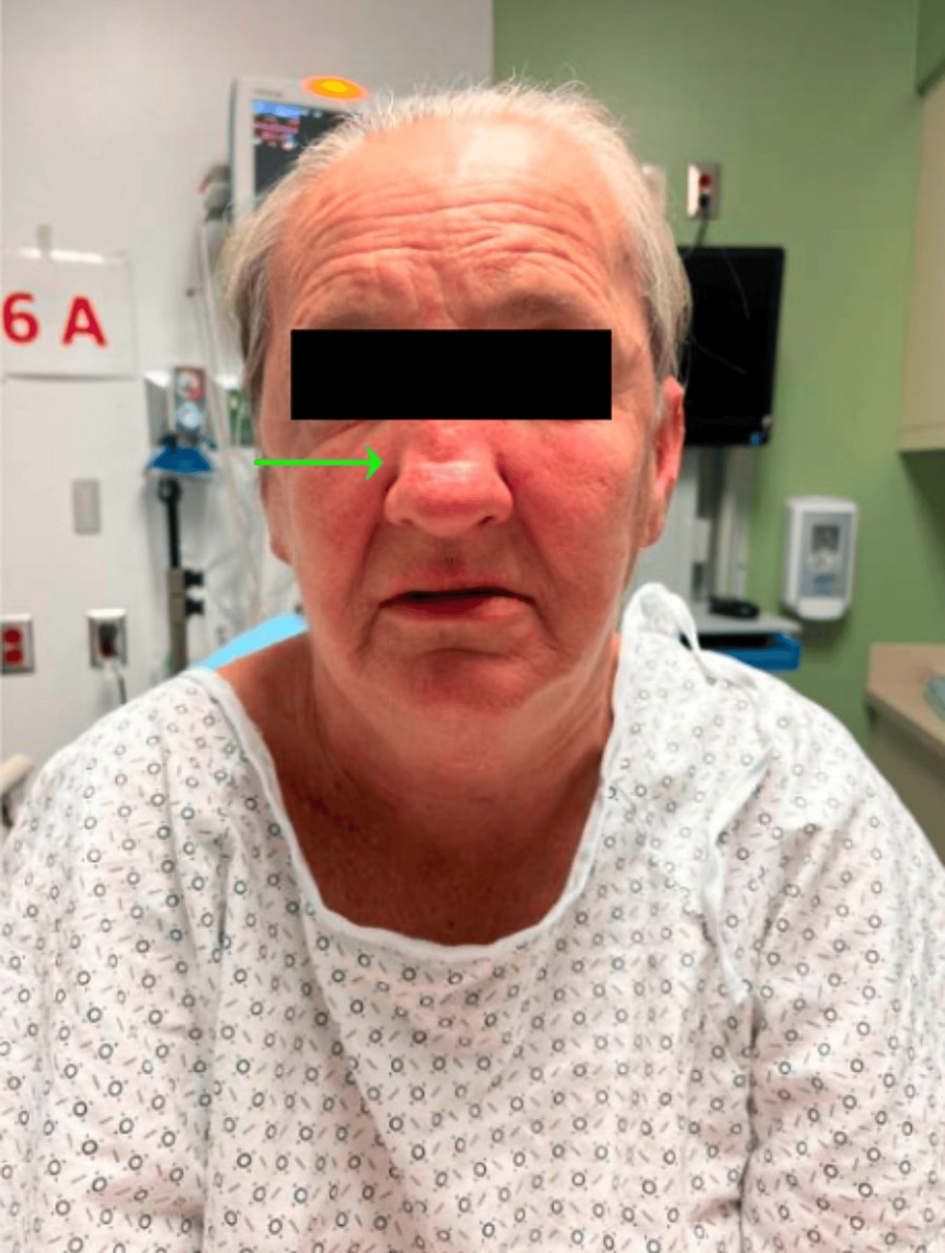
Notable asymmetric facial edema, left more than right, as well as a saddle nose

**Figure 2:**
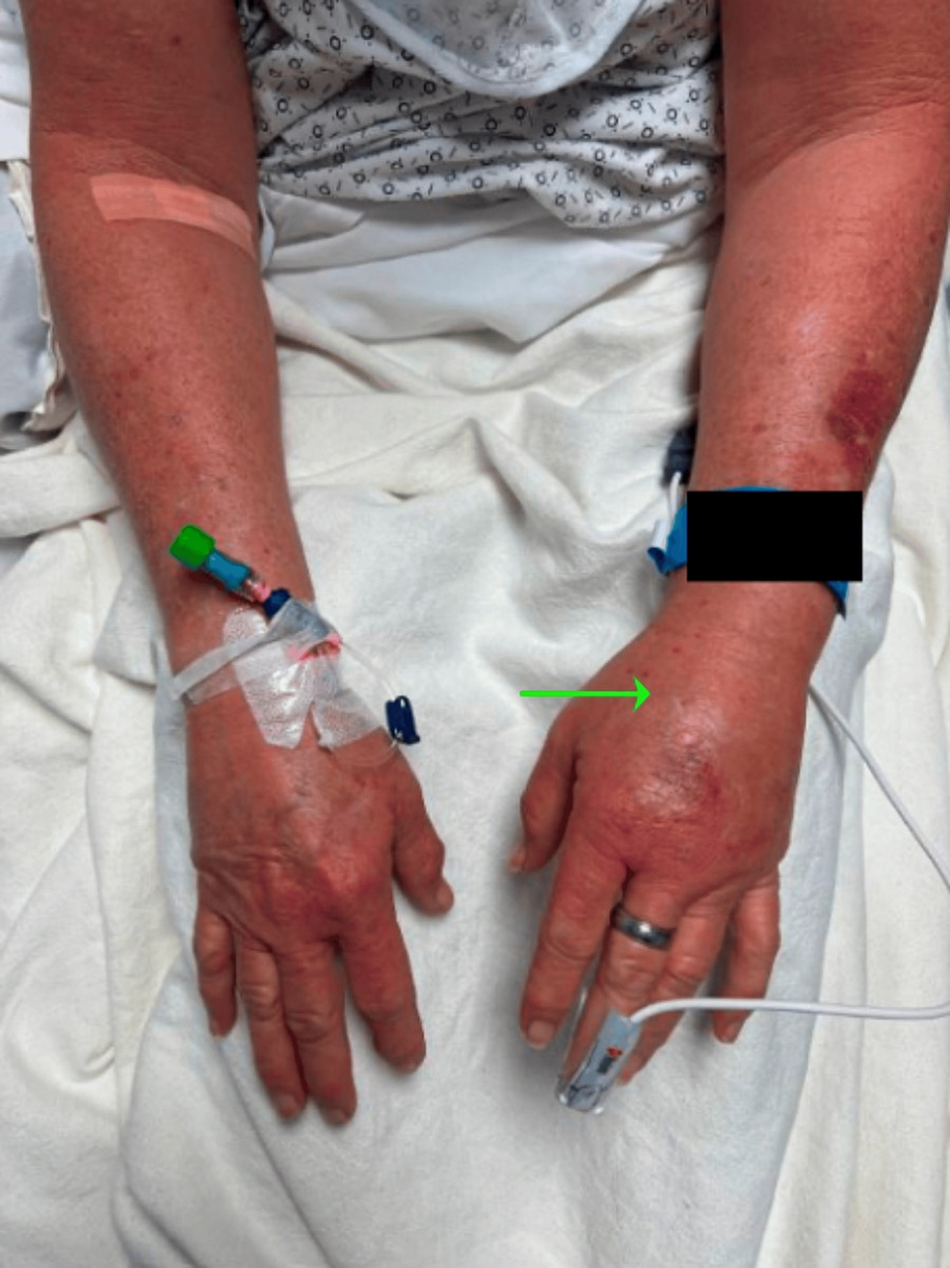
Upper extremity edema, left more than right

On presentation, her blood pressure was 156/68 mmHg, heart rate was 58 beats per minute, temperature was 98.0 degrees Fahrenheit, and respiratory rate was 19 breaths per minute with an oxygen saturation of 98% on room air. A physical exam revealed an anxious-appearing female with diffuse facial swelling and bilateral upper extremity swelling (see Figure 1 and Figure 2).

**Figure 3:**
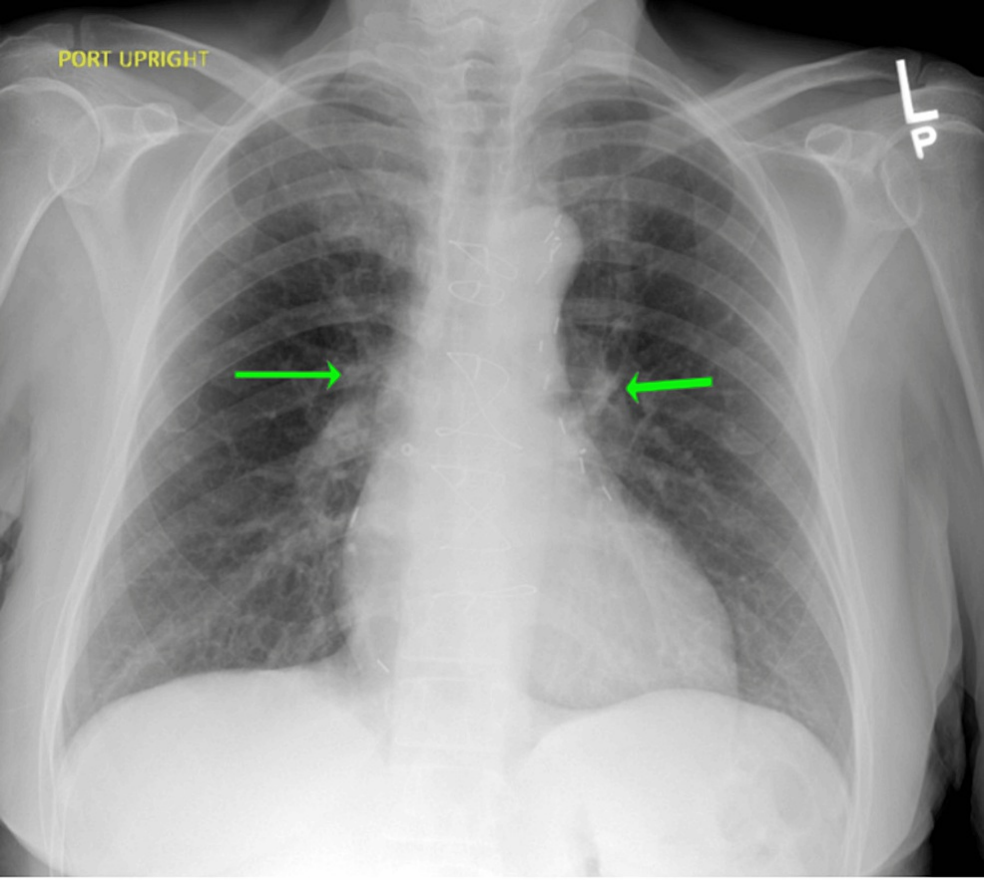
Chest radiograph anterior-posterior view showing no evidence of pulmonary infiltrates, pleural effusions, or pneumothorax. The heart size is in the upper limits of normal with evidence of previous cardiac surgery. There is prominence of central pulmonary vessels

**Figure 4:**
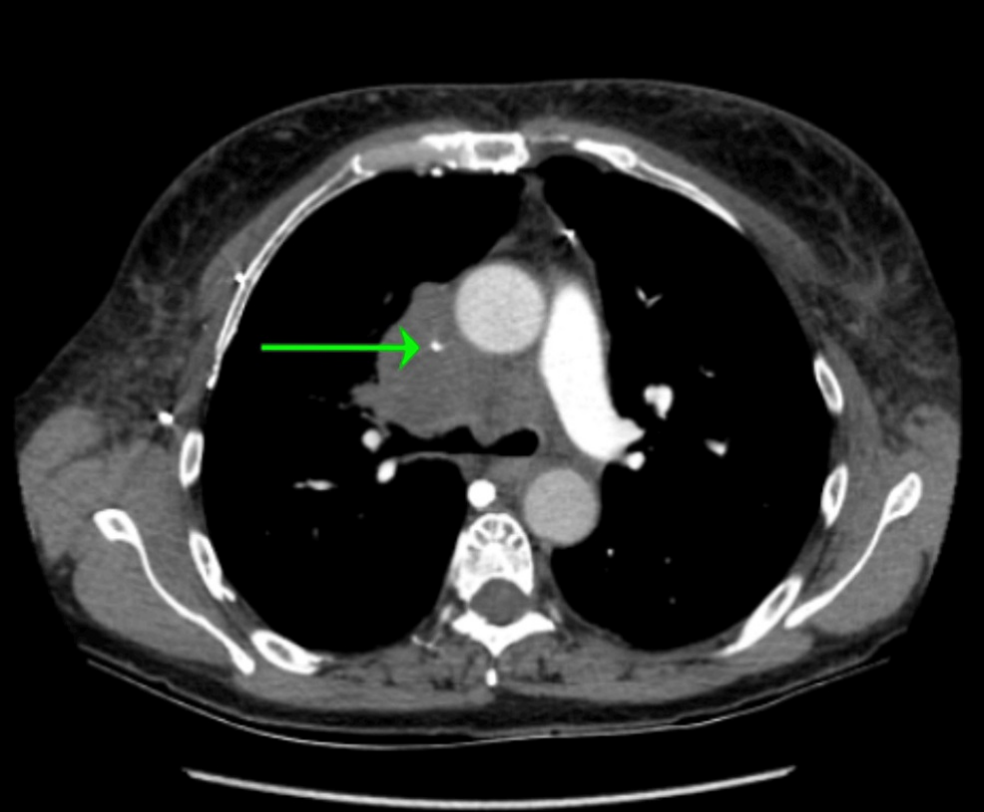
CTPA (axial view) showing severe narrowing of the SVC from compression by the right suprahilar mass CTPA: computed tomography pulmonary angiography; SVC: superior vena cava

**Figure 5:**
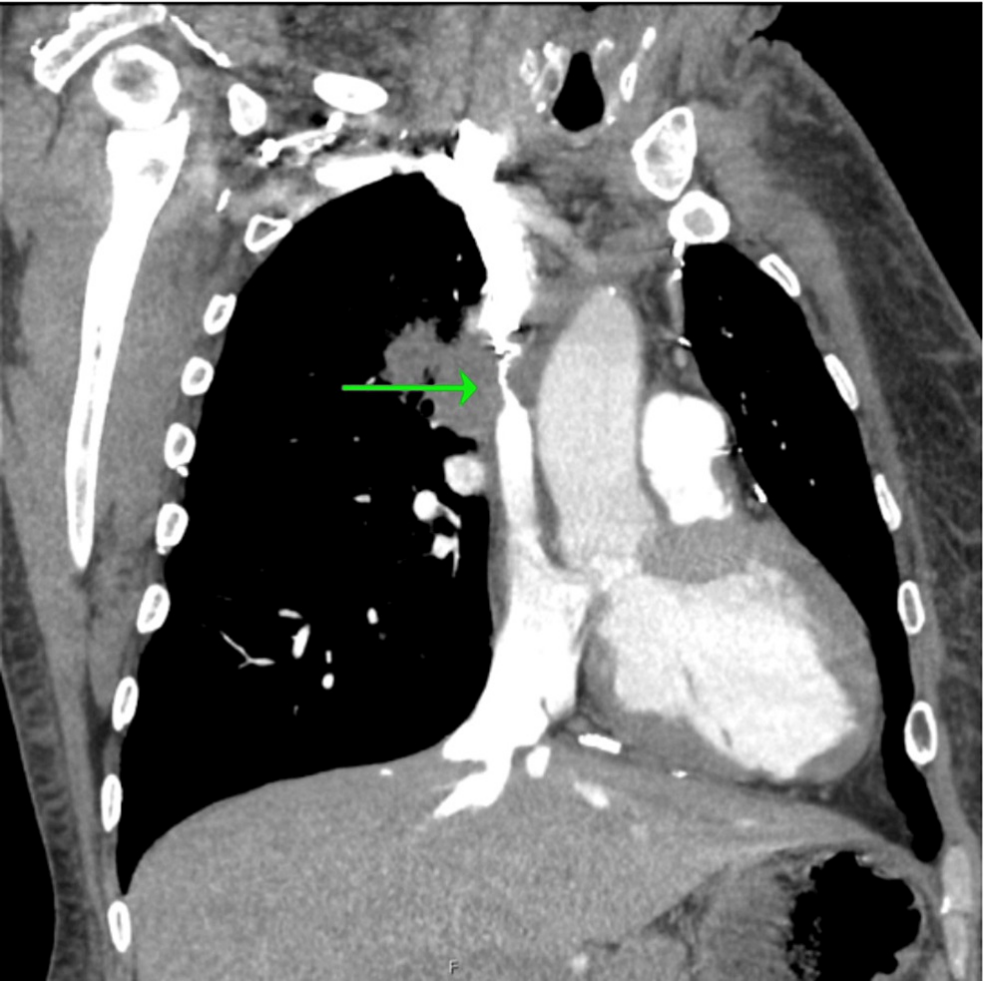
CTPA (coronal view) showing severe short segment narrowing of the SVC from compression by the right suprahilar mass CTPA: computed tomography pulmonary angiography; SVC: superior vena cava

**Figure 6:**
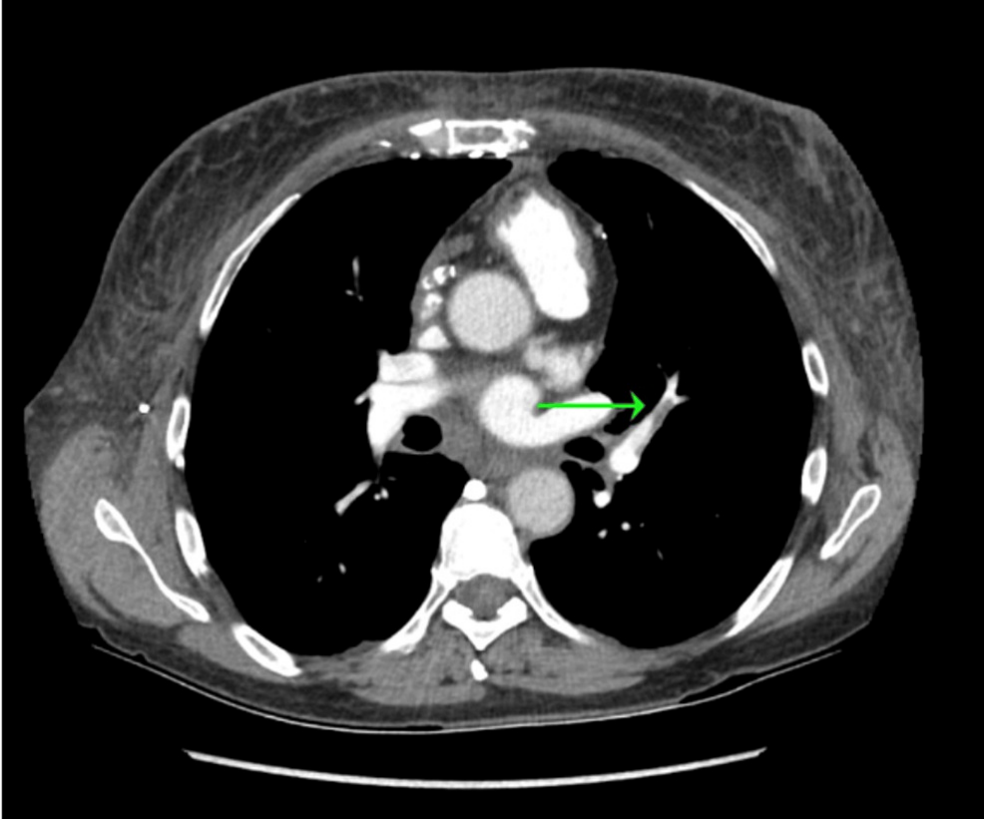
CTPA (axial view) showing subsegmental pulmonary emboli CTPA: computed tomography pulmonary angiography

Laboratory evaluation was largely unremarkable, with electrolytes, hemoglobin, and leukocytes within normal limits. A chest X-ray (CXR) radiography noted a central prominence of pulmonary vessels but was otherwise unremarkable (see Figure 3). A computed tomography pulmonary angiography (CTPA) revealed a right suprahilar mass measuring approximately 5.2 cm × 5.1 cm with severe compression of the right upper lobe pulmonary artery branch and severe short segment narrowing of the SVC (see Figure 4 and Figure 5). It also showed left upper and lower lobe segmental and subsegmental pulmonary emboli without right heart strain (see Figure 6).

**Figure 7:**
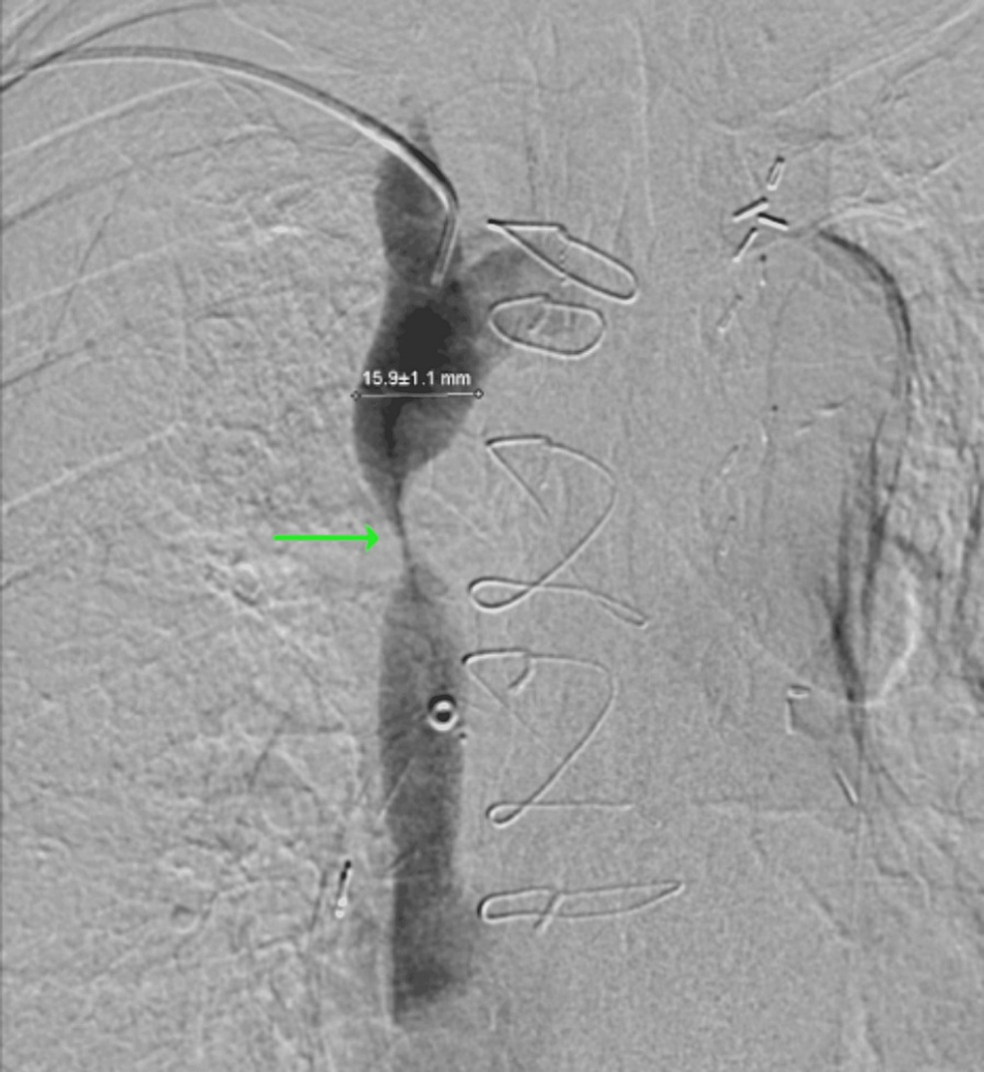
Venogram demonstrating SVC stenosis SVC: superior vena cava

**Figure 8:**
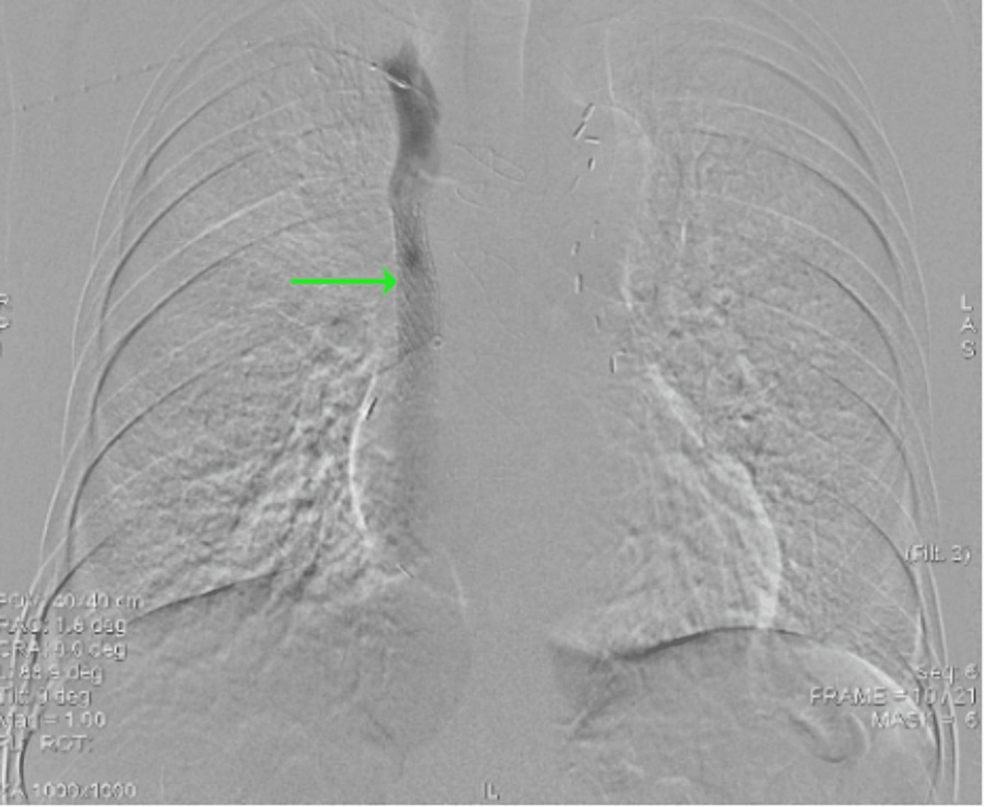
Venogram demonstrating the self-expandable Abre stent

**Figure 9:**
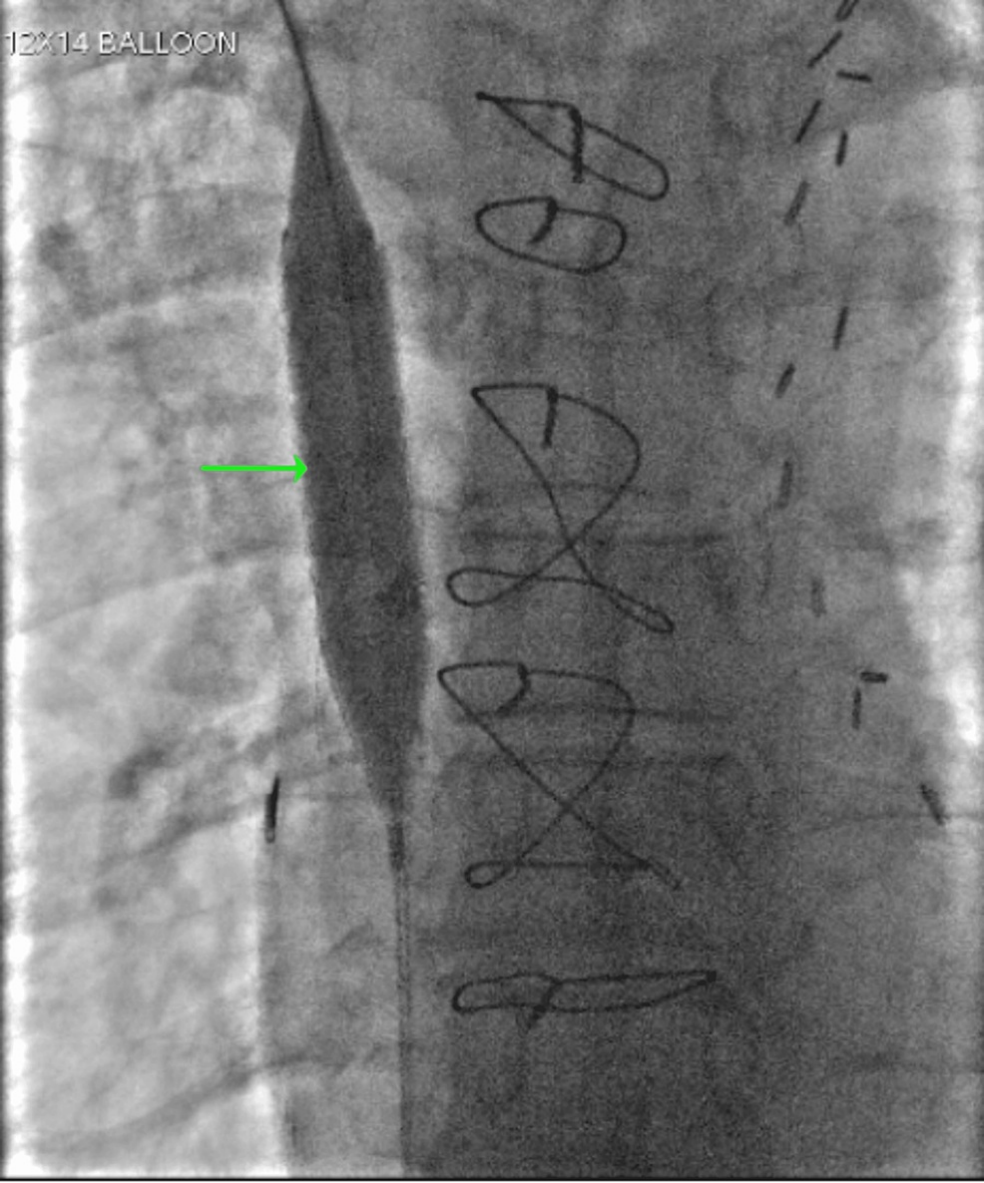
Venogram demonstrating the in-stent venoplasty with balloon

A heparin drip was subsequently started and interventional radiology was consulted for further management. An initial venogram demonstrated severe SVC stenosis related to the infiltrating right suprahilar mass (see Figure 7), so an intravascular biopsy of the mass using the HawkOne system was obtained for pathology review. Afterward, a 12 mm × 6 cm Abre stent was placed across the SVC stenosis below the level of brachiocephalic vein confluence and its distal portion at the cavoatrial junction (see Figure 8). This was followed by an in-stent venoplasty with 12 mm and 14 mm balloons (see Figure 9). An immunostain of the mass revealed positive synaptophysin, chromogranin, CD56, TTF1, and BCL-2 which was consistent with SCLC. The patient was transitioned to a direct oral anticoagulant and was discharged with near full resolution of her symptoms with plans for oncology follow-up outpatient.

## Discussion

SVC syndrome is diagnosed by clinical symptoms and advanced imaging. While malignancy is the most common cause, it is important to note that with more therapies requiring catheters ending in the right atrium, the incidence of nonmalignant causes is rising [4,5]. When obtaining a history from patients with common symptoms, screening for a history of malignancy and intravascular procedures is crucial [2]. Patients present with progressive symptoms, usually over several weeks, such as facial swelling, upper extremity swelling, distended neck veins, dyspnea, cough, and headaches [2,4,6]. In severe cases, confusion, visual changes, syncope, and coma can occur [4]. SVC syndrome that develops chronically can present with dilated tributary veins seen on physical exam [4].

Yu et al. proposed a grading scale based on clinical presentation, with grades ranging from 0 for asymptomatic to 5 for death [5]. Imaging with CXR is nonspecific and can show mediastinal widening and vascular engorgement. CT scans, such as the CTPA utilized in this patient, help characterize and localize the etiology of SVC obstruction. Venography is considered the gold standard for the diagnosis of SVC syndrome as collateral pathways are better visualized which helps guide management [4,7]. Compared to Yu et al., the Stanford method focuses on the anatomical obstruction visualized by venography and consists of four different types [4]. Its focus was to identify the patients at fatal risk of airway compromise who may need urgent intervention, but with patients having progressive symptoms, it is not as applicable [4]. The severity of presenting symptoms can guide therapy, along with the anatomic obstruction, but there is not yet an integrated classification that combines both [4] to further risk-stratify patients.

Initial management focuses on reducing symptoms with supplemental oxygen and stabilization of the airway. Elevating the head of the bed aims to reduce edema and decrease hydrostatic pressure [4]. Treatment is based on severity. If symptoms are not life-threatening and mass effect is suspected, patients can undergo tissue biopsy with staging evaluation to tailor chemotherapy with or without radiation [8]. However, if patients are at risk of circulatory compromise, they may require endovascular therapy including angioplasty, stenting, surgical bypass, and even SVC reconstruction [2,4]. In the case of our patient, she was a grade 1 or mild for edema in the head and neck based on Yu et al.'s symptom grading scale [9]; however, with the Stanford method, she was a type B with more than 90% stenosis prompting more urgent intervention [10]. The advantage of percutaneous stent placement is that it allows for symptomatic relief and an opportunity for interventional radiology to obtain a tissue biopsy. Obstruction due to intrinsic causes, such as thrombosis secondary to indwelling catheters, requires the removal of devices and consideration of anticoagulation [2,4].

Notably, this patient had presented to the ED one year before this admission for a chief complaint of chest pain, where a CTPA was performed and did not reveal any pulmonary emboli, lung mass, or lymphadenopathy. This aligns with the presentation of SCLC, which is known to be a rapidly growing malignancy with a doubling time estimated between 25 and 217 days [11]. This rapid growth poses a challenge for screening as SCLC is more often discovered past early, curative stages. It is important to note that our patient's CTPA was less sensitive than the CT of the chest for detecting lung cancer. A retrospective study done by Kino et al. showed that the rate of detection for lung cancer in patients presenting to the ED with pulmonary embolism through CTPA was low at 0.47% [12]. Therefore, the emphasis remains on guideline-directed screening through high-resolution CT for those with a smoking history [13]. 

## Conclusions

SVC syndrome is uncommon and should be diagnosed through careful history-taking, physical examination, and imaging. Since malignancy is the most common inciting event, clinicians should maintain a high index of suspicion when encountering patients with a history and symptoms consistent with SVC syndrome. Addressing the underlying cause is the mainstay of management, but more severe presentations may prompt endovascular therapy. Treatment should include a multidisciplinary team that may include internal medicine, radiology, surgery, and oncology. 
